# Insect pest control, approximate dynamic programming, and the management of the evolution of resistance

**DOI:** 10.1002/eap.1851

**Published:** 2019-02-12

**Authors:** Sean C. Hackett, Michael B. Bonsall

**Affiliations:** ^1^ Department of Zoology Mathematical Ecology Research Group South Parks Road Oxford OX1 3PS United Kingdom; ^2^ St. Peter's College New Inn Hall Street Oxford OX1 2DL United Kingdom

**Keywords:** agricultural insect pests, approximate dynamic programming, dynamic programming, look‐ahead policy, resistance management, stage structure, transgenic insect releases

## Abstract

Ecological decision problems, such as those encountered in agriculture, often require managing conflicts between short‐term costs and long‐term benefits. Dynamic programming is an ideal method for optimally solving such problems but agricultural problems are often subject to additional complexities that produce state spaces intractable to exact solutions. In contrast, look‐ahead policies, a class of approximate dynamic programming (ADP) algorithm, may attempt to solve problems of arbitrary magnitude. However, these algorithms focus on a temporally truncated caricature of the full decision problem over a defined planning horizon and as such are not guaranteed to suggest optimal actions. Thus, look‐ahead policies may offer promising means of addressing detail‐rich ecological decision problems but may not be capable of fully utilizing the information available to them, especially in scenarios where the best short‐ and long‐term solutions may differ. We constructed and applied look‐ahead policies to the management of a hypothetical, stage‐structured, continually reproducing, agricultural insect pest. The management objective was to minimize the combined costs of management actions and crop damage over a 16‐week growing season. The manager could elect to utilize insecticidal sprays or one of six release ratios of male‐selecting transgenic insects where the release ratio determines the number of transgenic insects to be released for each wild‐type male insect in the population. Complicating matters was the expression of insecticide resistance at non‐trivial frequencies in the pest population. We assessed the extent to which look‐ahead policies were able to recognize the potential threat of insecticide resistance and successfully integrate insecticides and transgenic releases to capitalize upon their respective benefits. Look‐ahead policies were competent at anticipating and responding to ecological and economic information. Policies with longer planning horizons made fewer, better‐timed insecticidal sprays and made more frequent transgenic releases, which consequently facilitated lower resistance allele frequencies. However, look‐ahead policies were ultimately inefficient resistance managers, and directly responded to resistance only when it was dominant and prevalent. Effective long‐term agricultural management requires the capacity to anticipate and respond to the evolution of resistance. Look‐ahead policies can accommodate all the information pertinent to making the best long‐term decision but may lack the perspective to actually do so.

## Introduction

Insect pests pose substantial challenges to both agriculture and public health through the direct consumption of agricultural produce and the vectoring of plant, livestock, and human pathogens. In addressing these challenges, economic and ecological considerations coincide: cost‐effective management practices must account for the ecological idiosyncrasies of the pest. This constrains the types of intervention that will be most useful and how the timing of those interventions should be planned. However, the selection pressure imposed by management interventions favors the survival of individuals expressing the capacity to tolerate or avoid control, facilitating the evolution of resistance. This adds an evolutionary component to questions of pest management that complicates the ease with which cost‐effective long‐term suppression can be achieved. There is an implicit discounting between our capacity to manage a pest today and to manage it in the future. Susceptibility to control is effectively a renewable resource that is depleted when we fail to moderate the strength of selection for resistance by accepting short‐term losses in exchange for possible longer‐term benefits (REX Consortium 2013, Mitchell and Onstad 2014).

In responding to the economic and ecological pressures of insect pest management it is rarely optimal to confront the focal pest with only a single management tool. For example, sterile insect releases can complement insecticidal sprays to achieve greater overall levels of suppression than when either control is used in isolation: in general, sprays are most effective when employed against a large population while the efficiency of sterile releases can increase as the target population declines (Barclay 2005). However, there has been a diversification of sterile insect methods in recent years, driven by developments in genetic engineering, resulting in novel approaches such as gene‐drive systems, male‐selecting strains, and sex ratio distortion (Bourtzis et al. 2016, Leftwich et al. 2016, Alphey and Bonsall 2017, Harvey‐Samuel et al. 2017). Male‐selecting strains are of particular relevance to resistance management. These insects carry a dominant lethal mutation that is benign when expressed in a male insect but deleterious when expressed in a female unless they are fed on a supplemented diet that compensates for the transgene (Alphey and Bonsall 2017). While male‐selecting releases have a reduced capacity for population suppression relative to a bi‐sex lethal release, the survival of the male offspring enables them to act as a source of alleles conferring susceptibility to other control measures such as insecticidal toxins. Thus, there exists the exciting potential for male‐selecting releases to complement existing control programs by permitting a proactive approach to resistance management while still contributing to population suppression (Alphey et al. 2007, 2009, Harvey‐Samuel et al. 2015, Zhou et al. 2018). However, the near‐term benefits of male‐selecting releases with respect to the prevention of crop damage are sensitive to the timing of lethality. If females are killed early then some damage will be averted relative to a scenario where no insects were released, but when lethality occurs late there will effectively be no immediate reductions in damage (Leftwich et al. 2016, Alphey and Bonsall 2017). Thus, effective integration of male‐selecting releases into pest management programs necessitates that the potential future benefits of a release can be assessed and weighted against more immediate costs.

The integration of distinct control options for the management of an insect pest population can be conceptualized as a dynamic program, a sequential problem where decisions are made recurrently and actions implemented now can influence the options and rewards available in the future (Powell 2014, Hackett and Bonsall 2018). For a given objective and set of actions, the iterative algorithmic technique of dynamic programming will identify the optimal mapping between possible states and actions. The algorithm proceeds by recursively identifying the action that maximizes the sum of both current and future benefits for each combination of time and state (Clark and Mangel 2000, Marescot et al. 2013). Thus, dynamic programming automatically accounts for the downstream consequences of decisions and has the desirable property of easily accommodating both stochasticity and nonlinearity (Marescot et al. 2013). However, the range of questions that dynamic programming can address is fundamentally limited by the curse of dimensionality. To operate, the algorithm must be able to compile the solution for each combination of state and time in a lookup table, which renders many complex, highly dimensioned problems intractable to this method (Powell 2014). This is unfortunate given the complexity inherent to many contemporary questions in insect pest management, particularly with regard to the delay or prevention of resistance evolution and the use of transgenic insect releases. While methods for reducing the dimensionality of a problem exist (Clark and Mangel 2000), such simplifications come at the expense of realism, which may be undesirable when it is the details of a problem (e.g., insect movement) that are of greatest interest.

This curse of dimensionality can be circumvented, and the range of questions available for exploration greatly expanded, by utilizing approximate dynamic programming (ADP), which provides a toolset for approaching problems of arbitrary complexity at the expense of guaranteed optimality (Powell 2011). That is, where dynamic programming would identify the best action, ADP will suggest a good action. Given that ecological decision problems frequently require the incorporation of numerous environmental, economic, and ecological processes this trade‐off may be justifiable (Nicol et al. 2010, Nicol and Chadès 2011). ADP encompasses four broad classes of policy: cost function approximations (CFAs), value function approximations (VFAs), policy function approximations (PFAs), and look‐ahead policies (Powell 2014, Powell and Meisel 2016).

Look‐ahead policies are distinguished from the other classes by their lack of reliance upon functional approximations. Instead, look‐ahead policies proceed by sequentially constructing and then explicitly solving a series of temporally truncated approximations of the full problem (Nicol et al. 2010, Powell 2014, Powell and Meisel 2016). The actions suggested by a look‐ahead policy are optimal only with respect to the truncated horizon (termed the planning horizon) over which they are solved and so are unlikely to be optimal with respect to the time horizon of the full problem. However, where the scale of the full problem renders it intractable, it may still be possible to identify effective actions by employing a look‐ahead policy with a sufficiently long planning horizon. While a comparatively brute‐force method, look‐ahead policies have several extremely valuable features: their runtime is independent of the magnitude of the state space, they are indifferent to non‐stationary parameters and they can be applied to questions where accurately approximating the downstream value of an action is problematic (Nicol et al. 2010, Powell 2011, 2014, Powell and Meisel 2016, Hackett and Bonsall 2018).

Look‐ahead policies potentially have much to offer to the study of ecological decision problems such as those in insect pest management. Their agnosticism to the complexity of the state space allows for the specification of models as complex as required, enabling them to represent granular details, which might otherwise need to be summarized, streamlined, or omitted (Nicol et al. 2010, Powell 2014). For example, look‐ahead policies can accommodate details such as how both wild‐type and released insects distribute themselves in space, how management actions unfold across this space (Garcia et al. 2016) and the timing and nature of insect dispersal and mating (Sudo et al. 2018). However, in addition to their near‐term consequences for pest population dynamics and the performance of management actions, these factors also have longer‐term implications for how resistance to control will evolve and spread (Téllez‐Rodríguez et al. 2014, Garcia et al. 2016, Sudo et al. 2018) that may not be properly accounted for by a basic look‐ahead policy. Furthermore, the limited temporal scope of look‐ahead policies may unduly bias them against utilizing controls such as male‐selecting releases that may entail accepting short‐term costs in exchange for longer‐term gains. The abridged nature of look‐ahead policies may also limit their capacity to adequately delay or reverse the evolution of resistance.

Here we apply look‐ahead policies to the management of a hypothetical agricultural insect pest with two life stages (juvenile and adult) and continuous reproduction. We consider the extent to which look‐ahead policies with planning horizons of different lengths are able to combine the use of insecticidal sprays and either early‐ or late‐acting male‐selecting transgenic releases to achieve population suppression. Additionally, we explore the capacity for look‐ahead policies to anticipate and respond to the threat of resistance evolution by initializing the pest population using insecticide resistance allele frequencies, which would be considered problematic in practice.

## Methods

### Overview

We explore the utility of look‐ahead policies for the integration of transgenic insect releases (comprising exclusively male insects carrying a dominant female lethal mutation, also referred to as a male‐selecting allele) and insecticidal sprays in the suppression of a hypothetical agricultural insect pest exhibiting non‐trivial levels of insecticide resistance. Furthermore, we highlight the implications of a cost‐minimizing approach for effective resistance management. That is, the presented model is not a resistance management model but a pest management model with pest resistance. Outcomes are contrasted for both early and late‐acting lethal transgenes. Notably, the model assumes perfect knowledge of a large number of variables and parameters. While dynamic programming methods, both exact and approximate, are extremely amenable to stochasticity and uncertainty (Clark and Mangel 2000, Nicol et al. 2010, Nicol and Chadès 2011, Marescot et al. 2013) our decision to keep the model deterministic enables ease of understanding and interpretation. However, these assumptions could usefully be relaxed to allow for stochastic effects that may influence the success or failure of an action such as precipitation. The possible influence of pest demography upon the selection of actions is considered by repeating all simulations for a pest population with a greatly reduced juvenile mortality rate in Appendix S1.

### Pest demography and genetics

We consider a hypothetical continuously reproducing agricultural insect pest population with a 1:1 sex ratio and a life cycle that can be broadly subdivided into two distinct stages: a non‐reproductive juvenile stage that directly damages the crop and a benign reproductive adult stage. Only adult insects are affected by management actions; juveniles cannot be mated to transgenic males and are rendered intractable to insecticides by being sequestered within, for example, the fruit or stem of the crop. An insect's genotype is determined by two independent genetic loci, each with two alternative alleles. One locus determines susceptibility to insecticide with alleles S (conferring susceptibility) and r (conferring resistance). The second locus represents the target for the lethal transgene with alleles w (denoting wild type) and L (the dominant lethal transgene). The transgene is assumed to function perfectly: no female insects inheriting the L allele survive to adulthood. Thus, male insects may possess any one of the following genotypes *g* = {SSww, Srww, rrww, SSwL, SrwL, rrwL}. Adult females may only possess the genotypes $g=\left\{\text{SSww,Srww,rrww}\right\}$. When the L allele is late acting, juvenile females with the genotypes $g=\left\{\text{SSwL,SrwL,rrwL}\right\}$ are viable but are killed before they attain maturity. Irrespective of the timing of lethality, released males always possess the genotype SSLL. Therefore, there are seven male genotypes to which adult females may be mated resulting in 21 distinct adult female stages *f* ^*i*×*j*^ where *i* denotes the maternal genotype and *j* the paternal genotype. Mating is instantaneous at the point of maturation and occurs uniformly and at random. All male genotypes are assumed to be equally competitive. Once mated, females do not mate again and produce juveniles at instantaneous rate λ.

Equal sex ratio allows explicitly tracking of only female numbers for all three ww genotypes and only male numbers for wL genotypes; the abundance of *ww* males is equivalent to the abundance of ww females and vice versa for female wL juveniles when considering late‐acting releases. Irrespective of their genotype, juveniles mature to adults at instantaneous rate σ and are subject to natural mortality at instantaneous rate μ_*j*_. Thus, changes in the abundance of each juvenile stage $j_{a^{g}}$ (where *a* denotes sex and *g* indicates genotype) are determined as

(1) $$\begin{array}{cc} \frac{dj_{f^{\mathrm{SSww}}}}{\mathrm{dt}} & =\lambda (f^{\mathrm{SSww}\times \mathrm{SSww}}\left(t\right)+0.5f^{\mathrm{SSww}\times \mathrm{Srww}}\left(t\right)\\ & \;+0.5f^{\mathrm{SSww}\times \mathrm{SSwL}}\left(t\right)\;+0.25f^{\mathrm{SSww}\times \mathrm{SrwL}}\left(t\right)\\ & \;+0.5f^{\mathrm{Srww}\times \mathrm{SSww}}\left(t\right)+0.25f^{\mathrm{Srww}\times \mathrm{Srww}}\left(t\right)\\ & \;+0.25f^{\mathrm{Srww}\times \mathrm{SSwL}}\left(t\right)+0.125f^{\mathrm{Srww}\times \mathrm{SrwL}}\left(t\right))\\ & \;-\mu_{j}j_{f^{\mathrm{SSww}}}\left(t\right)-\sigma_{j}j_{f^{\mathrm{SSww}}}\left(t\right) \end{array}$$

(2) $$\begin{array}{cc} \frac{dj_{f^{\mathrm{Srww}}}}{\mathrm{dt}} & =\lambda (0.5f^{\mathrm{SSww}\times \mathrm{Srww}}\left(t\right)+f^{\mathrm{SSww}\times \mathrm{rrww}}\left(t\right)\\ & \;+0.25f^{\mathrm{SSww}\times \mathrm{SrwL}}\left(t\right)+0.5f^{\mathrm{SSww}\times \mathrm{rrwL}}\left(t\right)\\ & \;+0.5f^{\mathrm{Srww}\times \mathrm{SSww}}\left(t\right)+0.5f^{\mathrm{Srww}\times \mathrm{Srww}}\left(t\right)\\ & \;+0.5f^{\mathrm{Srww}\times \mathrm{rrww}}\left(t\right)+0.25f^{\mathrm{Srww}\times \mathrm{SSwL}}\left(t\right)\\ & \;+0.25f^{\mathrm{Srww}\times \mathrm{SrwL}}\left(t\right)+0.25f^{\mathrm{Srww}\times \mathrm{rrwL}}\left(t\right)\\ & \;+f^{\mathrm{rrww}\times \mathrm{SSww}}\left(t\right)+0.5f^{\mathrm{rrww}\times \mathrm{Srww}}\left(t\right)\\ & \;+0.5f^{\mathrm{rrww}\times \mathrm{SSwL}}\left(t\right)+0.25f^{\mathrm{rrww}\times \mathrm{SrwL}}\left(t\right))\\ & \;-\mu_{j}j_{f^{\mathrm{Srww}}}\left(t\right)-\sigma_{j}j_{f^{\mathrm{Srww}}}\left(t\right) \end{array}$$

(3) $$\begin{array}{cc} \frac{dj_{f^{\mathrm{rrww}}}}{\mathrm{dt}} & =\lambda (0.25f^{\mathrm{Srww}\times \mathrm{Srww}}\left(t\right)+0.5f^{\mathrm{Srww}\times \mathrm{rrww}}\left(t\right)\\ & \;+0.125f^{\mathrm{Srww}\times \mathrm{SrwL}}\left(t\right)+0.25f^{\mathrm{Srww}\times \mathrm{rrwL}}\left(t\right)\\ & \;+0.5f^{\mathrm{rrww}\times \mathrm{Srww}}\left(t\right)+f^{\mathrm{rrww}\times \mathrm{rrww}}\left(t\right)\\ & \;+0.25f^{\mathrm{rrww}\times \mathrm{SrwL}}\left(t\right)+0.5f^{\mathrm{rrww}\times \mathrm{rrwL}}\left(t\right))\\ & \;-\mu_{j}j_{f^{\mathrm{rrww}}}\left(t\right)-\sigma_{j}j_{f^{\mathrm{rrww}}}\left(t\right) \end{array}$$

(4) $$\begin{array}{cc} \frac{dj_{m^{\mathrm{SSwL}}}}{\mathrm{dt}} & =\lambda (0.5f^{\mathrm{SSww}\times \mathrm{SSwL}}\left(t\right)+0.25f^{\mathrm{SSww}\times \mathrm{SrwL}}\left(t\right)\\ & \;+f^{\mathrm{SSww}\times \mathrm{SSLL}}\left(t\right)+0.25f^{\mathrm{Srww}\times \mathrm{SSwL}}\left(t\right)\\ & \;+0.125f^{\mathrm{Srww}\times \mathrm{SrwL}}\left(t\right)+0.5f^{\mathrm{Srww}\times \mathrm{SSLL}}\left(t\right))\\ & \;-\mu_{j}j_{m^{\mathrm{SSwL}}}\left(t\right)-\sigma_{j}j_{m^{\mathrm{SSwL}}}\left(t\right) \end{array}$$

(5) $$\begin{array}{cc} \frac{dj_{m^{\mathrm{SrwL}}}}{\mathrm{dt}} & =\lambda (0.25f^{\mathrm{SSww}\times \mathrm{SrwL}}\left(t\right)+0.5f^{\mathrm{SSww}\times \mathrm{rrwL}}\left(t\right)\\ & +0.25f^{\mathrm{Srww}\times \mathrm{SSLL}}\left(t\right)\;+0.25f^{\mathrm{Srww}\times \mathrm{SrwL}}\left(t\right)\\ & +0.25f^{\mathrm{Srww}\times \mathrm{rrwL}}\left(t\right)+0.5f^{\mathrm{Srww}\times \mathrm{SSLL}}\left(t\right)\\ & \;+0.5f^{\mathrm{rrww}\times \mathrm{SSwL}}\left(t\right)+0.25f^{\mathrm{rrww}\times \mathrm{SrwL}}\left(t\right)\\ & +f^{\mathrm{rrww}\times \mathrm{SSLL}}\left(t\right))\;-\mu_{j}j_{m^{\mathrm{SrwL}}}\left(t\right)\;-\sigma_{j}j_{m^{\mathrm{SrwL}}}\left(t\right) \end{array}$$

(6) $$\begin{array}{cc} \frac{dj_{m^{\mathrm{rrwL}}}}{\mathrm{dt}} & =\lambda (0.125f^{\mathrm{Srww}\times \mathrm{SrwL}}\left(t\right)+0.25f^{\mathrm{Srww}\times \mathrm{rrwL}}\left(t\right)\\ & \;+0.25f^{\mathrm{rrww}\times \mathrm{SrwL}}\left(t\right)\;+0.5f^{\mathrm{rrww}\times \mathrm{rrwL}}\left(t\right))\\ & \;-\mu_{j}j_{m^{\mathrm{rrwL}}}\left(t\right)-\sigma_{j}j_{m^{\mathrm{rrwL}}}\left(t\right) \end{array}$$

All adult stages are subject to both natural mortality μ_*a*_ and mortality from insecticide residues ϕ. The extent to which a given genotype (at the S/r locus) *g* tolerates exposure to insecticides is determined by δ^*g*^ where 0 ≤ δ^*g*^ ≤ 1 with δ^*g*^ = 0 indicating complete resistance (insects of genotype *g* are unaffected by exposure to insecticides) and δ^*g*^ = 1 indicating complete susceptibility (insects of genotype *g* experience the full effect of insecticicde exposure). For homozygotes, the value of δ^*g*^ is entirely determined by the insect's genotype. For Sr heterozygotes, δ^*g*^ is derived from the tolerances of homozygotes and the dominance of resistance *h* as

(7) $$\delta^{\mathrm{Sr}}=\left(1-h\right)\delta^{\mathrm{SS}}+h\delta^{\mathrm{rr}}$$

where 0 ≤ *h* ≤ 1 with *h* = 0 indicating fully recessive resistance and *h* = 1 indicating dominant resistance. We assume that resistance is not associated with any pertinent fitness costs.

The abundance of adult males heterozygous for the L allele is determined by iterative application of the following equation to each of the three possible transgenic heterozygote genotypes $g=\left\{\text{SSwL,SrwL,rrwL}\right\}$:

(8) $$\frac{dm^{g}}{\mathrm{dt}}=\sigma_{j}j_{m^{g}}\left(t\right)-\left(\mu_{a}+\delta^{g}\varphi \left(t\right)\right)m^{g}\left(t\right).$$

Given the equal sex ratio, the total abundances of SSww, Srww, and rrww adult males are derived from the abundance of adult females with the corresponding genotype as $m^{j}=\sum_{i}f^{i\times j}$. The total number of males available for mating *M* is found as the sum of all male genotypes excluding released males, *m* ^SSLL^. Thus, adult females are allocated between mated stages according to

(9) $$\frac{df^{i\times j}}{\mathrm{dt}}=\left(\frac{m^{j}\left(t\right)}{M\left(t\right)}\right)\sigma_{j}j_{f^{a}}\left(t\right)-\left(\mu_{a}+\delta^{i}\varphi \left(t\right)\right)f^{i}\left(t\right)$$

where the indices *i* and *j* denote male and female genotype, respectively.

### Actions and costs

The management objective is to minimize the cumulative costs of both pest induced losses and pest management over the duration of a single season. The season is comprised of *W* = 16 discrete weeks (a broad representation of a temperate crop season) and a single action may be implemented at the start of each week *w*. Actions are selected with reference to the current state of the system *S*_*w*_, which is defined as the minimally dimensioned function of history required to model the transitions of the system from a given point in time onward (Powell 2011). Once implemented, an action instantaneously updates the pertinent control variable and the manager experiences a point cost. Insecticide application is uniform across the landscape and sets φ, the instantaneous rate of mortality from insecticide, to its highest value φ_max_ while incurring the cost *c*_spray_. Subsequent to application, pesticide toxicity decays exponentially

(10) $$\frac{d\varphi }{\mathrm{dt}}=-\alpha \varphi \left(t\right)$$

where α determines the persistence of the pesticide. For transgenic releases, six alternative release ratios are available (1:1, 2:1, 5:1, 10:1, 15:1 and 20:1). The selected release ratio dictates the number of transgenic males to be released per wild‐type male. For example, selecting a release ratio of 10:1 will see a number of transgenic males released equal to 10 times the number of wild‐type males that were in the target population at the time the decision was made. Thus, the cost of a release *c*_rel_ varies with the abundance of non‐transgenic males and the release ratio. Once released, transgenic male abundance declines according to

(11) $$\frac{dm^{\mathrm{SSLL}}}{\mathrm{dt}}=-\left(\mu_{a}+\delta^{\mathrm{SS}}\varphi \left(t\right)\right)m^{\mathrm{SSLL}}\left(t\right)$$

which assumes that the transgenic males are equivalent to wild‐type males and experience no ill effects from carrying two copies of the lethal construct.

Costs inflicted by juvenile insects accrue as the product of total juvenile abundance (across both sexes and all genotypes) and the rate parameter γ, which proxies the susceptibility of the crop to feeding damage, with greater values implying greater susceptibility. Thus, the increase in costs caused by larval feeding for late‐acting releases is found as

(12) $$\frac{\mathrm{dc}}{\mathrm{dt}}=\gamma \left(2\sum_{i}{j_{f}}^{i}+2\sum_{j}j_{m^{j}}\right)$$

where *j*_*f*_ refers to the abundance of female ww homozygotes ($i=\left\{\text{SSww,Srww,rrww}\right\}$) and *j*_*m*_ denotes the abundance of male wL heterozygotes ($\;j=\left\{\text{SSwL,SrwL,rrwL}\right\}$). These values are doubled for late‐acting lethality to account for the abundance of the opposite sex. For early‐acting lethality, there are no female wL juveniles and it is necessary to double only *j*_*f*_.

Eqs. (1), (2), (3), (4), (5), (6), (7), (8), (9), (10), (11), (12) define the transition function for the model and how the state of the system changes over time in response to management actions (Powell 2014, Powell and Meisel 2016). Given Eqs. (1), (2), (3), (4), (5), (6), (7), (8), (9), (10), (11), (12) and the definition of the state of the system as the minimally dimensioned function of history required to model a system from a given point in time onward, the state of the current model at the onset of any given week *w* may be formally summarized as

(13) $$\begin{array}{cc} S_{w} & =\left\{j_{f^{\mathrm{SSww}}}\left(t\right),j_{f^{\mathrm{Srww}}}\left(t\right),j_{f^{\mathrm{rrww}}}\left(t\right),j_{m^{\mathrm{SSwL}}}\left(t\right),j_{m^{\mathrm{SrwL}}}\left(t\right),j_{m^{\mathrm{rrwL}}}\left(t\right),\right.\\ & f^{\mathrm{SSww}\times \mathrm{SSww}}\left(t\right),f^{\mathrm{SSww}\times \mathrm{Srww}}\left(t\right),f^{\mathrm{SSww}\times \mathrm{rrww}}\left(t\right),f^{\mathrm{SSww}\times \mathrm{SSwL}}\left(t\right),\\ & f^{\mathrm{SSww}\times \mathrm{SrwL}}\left(t\right),f^{\mathrm{SSww}\times \mathrm{rrwL}}\left(t\right),f^{\mathrm{SSww}\times \mathrm{SSLL}}\left(t\right),\\ & f^{\mathrm{Srww}\times \mathrm{SSww}}\left(t\right),f^{\mathrm{Srww}\times \mathrm{Srww}}\left(t\right),f^{\mathrm{Srww}\times \mathrm{rrww}}\left(t\right),f^{\mathrm{Srww}\times \mathrm{SSwL}}\left(t\right),\\ & f^{\mathrm{Srww}\times \mathrm{SrwL}}\left(t\right),f^{\mathrm{Srww}\times \mathrm{rrwL}}\left(t\right),f^{\mathrm{Srww}\times \mathrm{SSLL}}\left(t\right),f^{\mathrm{rrww}\times \mathrm{SSww}}\left(t\right),\\ & f^{\mathrm{rrww}\times \mathrm{Srww}}\left(t\right),f^{\mathrm{rrww}\times \mathrm{rrww}}\left(t\right),f^{\mathrm{rrww}\times \mathrm{SSwL}}\left(t\right),f^{\mathrm{rrww}\times \mathrm{SrwL}}\left(t\right),\\ & f^{\mathrm{rrww}\times \mathrm{rrwL}}\left(t\right),f^{\mathrm{rrww}\times \mathrm{SSLL}}\left(t\right),m^{\mathrm{SSwL}}\left(t\right),m^{\mathrm{SrwL}}\left(t\right),m^{\mathrm{rrwL}}\left(t\right),\\ & \left.m^{\mathrm{SSLL}}\left(t\right),\varphi \left(t\right),c\left(t\right)\right\}. \end{array}$$

Given the stated objective of cost minimization, for any given week the optimal action $a_{w}^{*}$ is that which satisfies

(14) $$a_{w}^{*}=\arg\min_{a_{w}}\left\{C\left(S_{w},a_{w}\right)+\sum_{w^{\prime }=w+1}^{W}\left(C\left(S_{w^{\prime }},a_{w^{\prime }}\right)\right)\right\}$$

where $C\left(S_{w},a_{w}\right)$ is the contribution function, which determines the full cost accruing between week *w* and week *w* + 1 as

(15) $$C\left(S_{w},a_{w}\right)=c_{a}+\int_{t^{\prime }=0}^{t^{\prime }=t_{w}}2\gamma l\left(t^{\prime }\right)$$

where *c*_*a*_ denotes the point cost for the action implemented ($c_{\mathrm{spray}},c_{\mathrm{rel}},$ or 0 in the case of inaction) at the start of week *w* and 2γ*l*(*t*′) is equivalent to Eq. (12). For a sufficiently small state space, Eq. (14) could be solved exactly using dynamic programming (Marescot et al. 2013). The solution would be to compile a lookup table detailing the optimal combinations of action and state for each permissible instance of the state for each decision period (Clark and Mangel 2000, Marescot et al. 2013). However, the state space for the current problem is both large and continuously valued (Eq. (13)) necessitating the use of alternative methods.

### Decision making

We choose to approach this problem using a look‐ahead policy, a class of approximate dynamic programming algorithm that is indifferent to the dimensionality of the state space and forgoes attempts to solve Eq. (14), instead solving a series of temporally truncated approximations of Eq. (14) to suggest actions that, while suboptimal, may be “good enough” (Nicol et al. 2010, Powell 2011, 2014). The problem we seek to solve, Eq. (14) is termed the base model while the truncated approximations solved in its place are termed look‐ahead models (Powell 2014, Powell and Meisel 2016). A look‐ahead policy proceeds forward in time, from the current week *w* up to the time horizon *W*. For each week, a best action *a*_*w*_ is identified by solving a look‐ahead model over an interval of time spanning from *w*, the current week, up to some *H* < *W*, where *H* is a planning horizon that determines the number of decision periods into the future the model will “look ahead,” as follows:

(16) $$a_{w}=\arg\min_{a_{w}}\left\{C\left(S_{w},a_{w}\right)+\sum_{w^{\prime }=w+1}^{w^{\prime }=w+H}\left(C\left(S_{ww^{\prime }}^{∼},a_{ww^{\prime }}^{∼}\right)\right)\right\}$$

where tildes distinguish values pertaining to the look‐ahead model from those in the base model. Thus, $S_{ww^{\prime }}^{∼}$ is the state experienced during week *w*′ when solving a look‐ahead model to identify an action for week *w*. Eq. (16) states that the best action, with respect to the look‐ahead model, is that which minimizes cumulative costs over the interval $\left(w,\ldots ,w+H\right)$. This is not guaranteed to be the same action that would minimize costs over the full interval of the base model $\left(w,\ldots ,W\right)$ given the look‐ahead models incomplete knowledge of the downstream effects of an action. Solving Eq. (16), a look‐ahead model, is not equivalent to solving Eq. (14), the base model. However, by solving Eq. (16) for a planning horizon of sufficient length to capture important behaviors, actions can be suggested that will outperform those recommended by a purely myopic policy that minimizes only the immediate costs of an action (Nicol et al. 2010, Powell 2011, 2014, Powell and Meisel 2016, Hackett and Bonsall 2018). Actions taken within the look‐ahead model are effectively exploratory, serving only to improve the quality of the current decision. Thus, once *a*_*w*_, the action to be taken now, has been identified states and actions experienced as part of the look‐ahead model are discarded. Action *a*_*w*_ is then implemented and the model transitions to *S*_*w*+1_ where a new look‐ahead model is constructed and solved over the interval $\left(w+1,\ldots ,w+1+H\right)$ to return action *a*_*w*+1_.

### Simulations

Simulations were performed using code written in R version 3.3.3. The pest population is initiated with 10,000 mated adult females, 10,000 wild‐type adult males, and one of three insecticide resistance allele frequency of *r*_0_ = {0.3, 0.5, 0.7}. The initial frequency of the resistance allele is then used to divide the founding females between the SSww, Srww, and rrww genotypes. The lethal transgene is assumed to be absent at start of the season. Given a 1:1 sex ratio, females are subsequently allocated to mated classes based on the relative abundance of each genotype at the S/r locus. Performance was evaluated for a myopic policy (*H *= 0) and three look‐ahead policies *H* = {1,2,3}. For each policy, simulations were repeated for three levels of genetic dominance of resistance *h* = {0, 0.5, 1.0} (describing recessive, additive and dominant resistance respectively), two levels of natural juvenile mortality $\mu_{j}=\left\{0.2,0.05\right\}$ (which we term the default and robust populations, respectively) and for both early‐ and late‐acting releases. Continuous time dynamics were approximated using a fourth‐order Runge‐Kutta (RK4) solver with a resolution of *t*_*w*_ = 700 time increments. Values for all model parameters are summarized in Table 1.

**Table 1:** Summary of model parameters and their meanings

Parameter	Meaning	Default value
λ	female eggs laid per adult female per day	1.0
μ_*j*_	instantaneous juvenile natural mortality rate	default = 0.2, robust = 0.05
μ_*a*_	instantaneous adult natural mortality rate	0.05
σ_*j*_	instantaneous juvenile maturation rate	0.1
*h*	dominance of resistance	0,0.5,1.0
δ^SS^	susceptibility of an SS adult to insecticide	1.0
δ^Sr^	susceptibility of an Sr adult to insecticide	(1 − *h*)*δ* ^SS^ + *h*δ^rr^
δ^rr^	susceptibility of an rr adult to insecticide	0
*c* ^spray^	point cost of insecticide application	4,000
*c* ^ste^	point cost per self‐limiting male released	0.003
α	instantaneous insecticide decay rate	0.35
ϕ_max_	maximum instantaneous insecticide mortality rate	0.9
γ	instantaneous rate at which costs/losses from juvenile feeding accrue	0.01
*W*	season length (weeks)	16
*t*_*w*_	resolution of the fourth‐order RK4 solver	700

## Results

**Figure 1:**
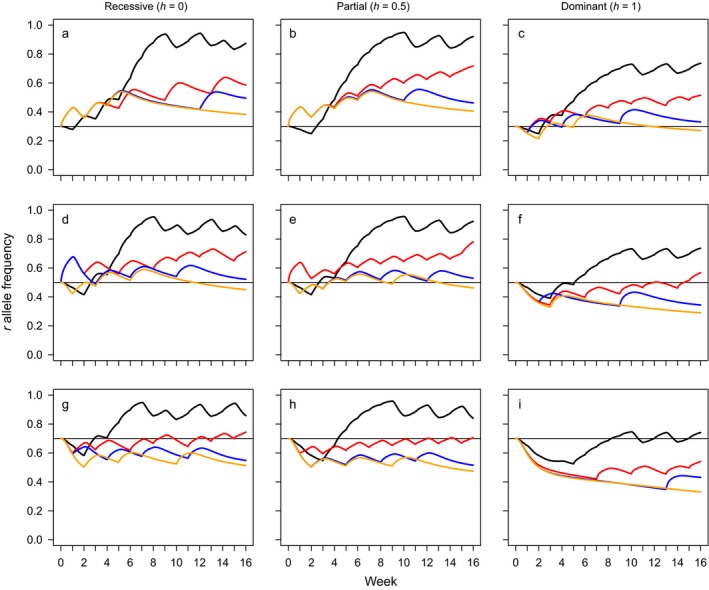
Insecticide resistance allele trajectories for four alternative policies attempting to manage a hypothetical insect pest over a 16‐week season using insecticidal sprays and releases of transgenic male insects carrying a late‐acting male‐selecting transgene. Black lines depict trajectories for a myopic policy with a planning horizon of *H* = 0 while red, blue, and orange lines portray look‐ahead policies with planning horizons of *H* = 1, *H* = 2, and *H* = 3, respectively. For all policies, *H* is measured in weeks. All panels within the same row share the same initial resistance allele frequency *r*_0_ (indicated by a horizontal black line, from $r_{0}=0.3\left(a,b,c\right)$, to $r_{0}=0.5\left(d,e,f\right)$ to $r_{0}=0.7\left(g,h,i\right)$.), and all panels within the same column share the same dominance of resistance *h* (completely recessive resistance, $h=0\left(a,d,g\right)$; additive resistance, $h=0.5\left(b,e,h\right)$; dominant resistance, $h=1\left(c,f,i\right)$).

Myopic policies $\left(H=0\right)$ were consistently outperformed by look‐ahead policies $\left(H\geq 1\right)$ across all scenarios with respect to cost minimization. Additionally, for the default pest population $\left(\mu_{j}=0.2\right)$ look‐ahead policies with longer planning horizons outperformed those with shorter ones irrespective of the initial resistance allele frequency *r*_0_ and the dominance of resistance *h*. For the same initial conditions, look‐ahead policies with longer planning horizons suggest actions that provide better cost reductions and lower resistance allele frequencies. This is illustrated in Fig. 1, which shows the change in the frequency of the insecticide resistance allele over the duration of the season for all four policies.

Instances of spraying can be discerned in Fig. 1 from the increases in the frequency of resistance alleles that they induce. Similarly, transgenic releases can be identified by declines in resistance allele frequency. Notably, for all presented combinations of *r*_0_ and *h*, policies with longer planning horizons make less frequent use of sprays than shorter ones. Policies with longer planning horizons have an improved capacity to weight the long‐term benefits of a well‐timed release against the short‐term costs from feeding by both male and female transgenic heterozygote juveniles. Intuitively, the frequency of sprays is also sensitive to the initial resistance allele frequency and the dominance of the resistance allele. In general, partially dominant resistance incentivizes spraying, particularly for the myopic policy and the shortest look‐ahead policy (*H* = 1). Conversely, dominant resistance discourages spraying and promotes additional releases, especially for look‐ahead policies with longer planning horizons of $H=\left\{2,3\right\}$ and this effect increases with the initial frequency of the resistance allele (e.g., compare Fig. 1c, i). The increase in the frequency of spraying when resistance is partially dominant highlights the value of insecticidal sprays to the model: sprays provide an immediate decrease in adult abundance and thus a reduction in the production of juveniles. In contrast, late‐acting transgenic releases do not reduce the extant number of reproductive adult females, they instead restrict the future recruitment of adult females. Furthermore, when releases are late acting, females inheriting the lethal allele will still cause feeding damage. Thus, given the stated objective of cost minimization, the model tends to respond to reductions in spray efficacy with an increase in spraying effort and will only make a concerted switch toward transgenic releases as the sole method of suppression when spray efficacy degrades significantly.

**Figure 2:**
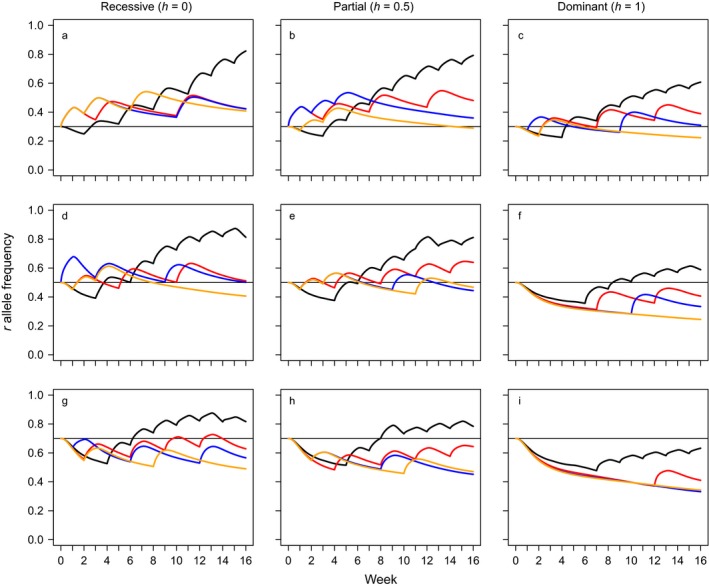
Insecticide resistance allele trajectories for four alternative policies attempting to manage a hypothetical insect pest over a 16‐week season using insecticidal sprays and releases of transgenic male insects carrying an early‐acting male‐selecting transgene. Black lines depict trajectories for a myopic policy with a planning horizon of *H* = 0 while red, blue, and orange lines portray look‐ahead policies with planning horizons of *H* = 1, *H* = 2, and *H* = 3, respectively. For all policies, *H* is measured in weeks. All panels within the same row share the same initial resistance allele frequency *r*_0_ (indicated by a horizontal black line, from $r_{0}=0.3\left(a,b,c\right)$, to $r_{0}=0.5\left(d,e,f\right)$ to$r_{0}=0.7\left(g,h,i\right)$.), and all panels within the same column share the same dominance of resistance *h* (completely recessive resistance, $h=0\left(a,d,g\right)$; additive resistance, $h=0.5\left(b,e,h\right)$; dominant resistance, $h=1\left(c,f,i\right)$).

**Figure 3:**
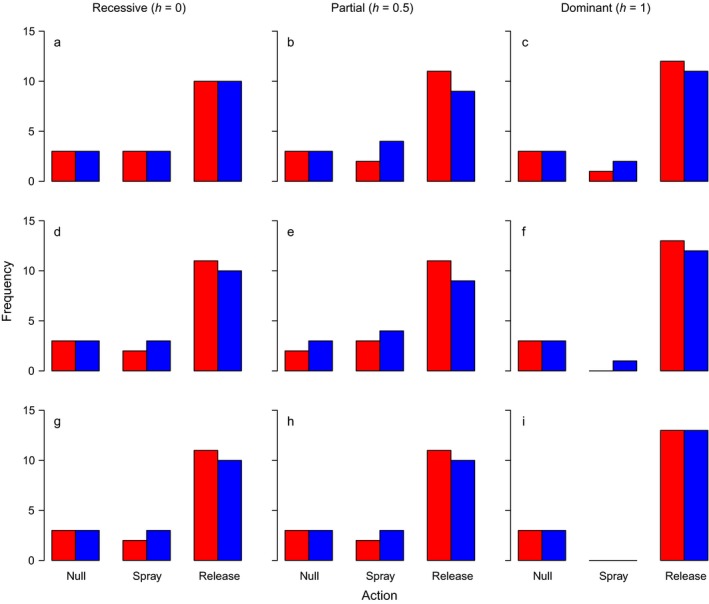
Frequencies of implemented actions over a 16‐week season for a look‐ahead policy with a planning horizon of *H* = 3 weeks. In a given week, the model may select to do nothing (null), apply foliar insecticide (spray), or choose from one of six transgenic male insect release ratios (here aggregated together as one category: release). Red bars, transgenic males carry an early‐acting female lethal construct; blue bars, construct is late acting. All panels within the same row share the same initial resistance allele frequency *r*_0_ (indicated by a horizontal black line), and all panels within the same column share the same dominance of resistance *h*. Moving from the top row to the bottom row the initial resistance allele frequency increases from $r_{0}=0.3\left(a,b,c\right)$, to $r_{0}=0.5\left(d,e,f\right)$ to $r_{0}=0.7\left(g,h,i\right)$. Panels in the left column illustrate results for completely recessive resistance $h=0\left(a,d,g\right)$, panels in the central column show results for additive resistance $h=0.5\left(b,e,h\right)$, while panels in the right column show results for dominant resistance $h=1\left(c,f,i\right)$.

The general trends observed for late‐acting releases also hold true for early‐acting releases, which produce qualitatively similar outcomes to late‐acting releases with respect to resistance allele frequency (Fig. 2). However, while the end point is similar, for any given combination of *h* and *r*_0_, a lesser cost is incurred and the action sequences employed are distinct from those utilized for late‐acting releases. By removing juvenile females from the population before they feed, early‐acting releases confer a partial reduction in feeding damage at the point of use in addition to reducing future reproduction. Consequently, sprays are used less frequently when releases are early acting. This is illustrated in Fig. 3, which shows the frequency with which each action category (no action, spray, or release of transgenics) was selected for the look‐ahead policies with planning horizons of *H* = 3 depicted in Fig. 1 (late‐acting, blue) and Fig. 2 (early acting, red). These trends also apply when the natural juvenile mortality rate is reduced (μ_*j*_ = 0.05, the robust population) with the exception that the increase in juvenile survivability promotes additional instances of insecticide spraying (Appendix S1: Figs. S1 and S2).

Irrespective of the type of release, and in spite of the reductions in resistance allele frequency observed for longer planning horizons, it is notable that no policy makes an intensive effort to eradicate resistance. The actions selected by a given policy result in a decline in pest abundance over the season (albeit via different means and over different time scales), which is a desirable outcome with respect to the cost‐minimizing objective function as fewer pests entails less feeding damage. However, given that actions carry a cost and the damage is inflicted upon the crop in proportion to juvenile abundance it follows that the impetus for an action to be selected decreases as the pest population declines. Furthermore, the focus upon a single season entails that there is no penalty to ending the season with a high resistance allele frequency as the value of insecticides in the subsequent season is not accounted for. Thus if, due to actions taken during the early weeks of the season, the pest population has been significantly reduced but resistance has become prevalent then no action will be taken to mitigate this: the effects of a largely resistant population in the following season are not pertinent to decision making. The policies presented here are only incentivized to concentrate upon reducing the resistance allele frequency when the potential for damage is high and susceptibility to sprays is low. This can be observed in Figs. 1 and 2: when resistance is initially prevalent (e.g., *r*_0_ ≥ 0.5) and particularly when it is non‐recessive (so some heterozygotes may survive insecticide exposure) then most policies will reduce the resistance allele frequency to some extent prior to utilizing sprays. Thus, while look‐ahead policies with longer planning horizons tend to produce lower resistance allele frequencies they are not explicitly managing resistance. Instead, they are utilizing their improved capacity to resolve how the consequences of actions unfold over time to select actions such that costs are minimized, an approach that, incidentally, favors fewer instances of spraying and more transgenic releases.

## Discussion

We applied a class of approximate dynamic programming algorithms, the look‐ahead policy, which identifies useful actions by solving a truncated version of the full optimization problem, to the management of a simple, deterministic representation of a continuously reproducing, stage‐structured agricultural insect pest. Look‐ahead policies consistently suggested sequences of decisions that provided lower costs and resistance allele frequencies than a myopic policy (which optimizes only with respect to a single time period). These benefits were made available by the superior capacity of look‐ahead policies to integrate insecticidal sprays and male‐selecting transgenic insect releases and could be improved upon by extending the planning horizon, *H* (Figs. 1, 2; Appendix S1: Fig. S1). This was true for both early‐ and late‐acting transgenic releases although, intuitively, the lowest costs and resistance allele frequencies were reported for early‐acting releases, which also made fewer applications of insecticide (Fig. 3; Appendix S1: Fig. S2). Look‐ahead policies were also shown to be able to adjust their decisions to accommodate significant changes to the demography of the pest (Appendix S1: Figs. S1 and S2). However, while these findings indicate that approximate optimization methods such as the look‐ahead policy may be a valuable tool in addressing complex ecological decision problems (Nicol et al. 2010, Nicol and Chadès 2011) there is significant room for improvement if these approaches are to be gainfully applied to problems with an evolutionary component, where the longer‐term responses of the target population must be anticipated (Ashley et al. 2003, Brown and Staňková 2017). Given an adequate planning horizon, unmodified look‐ahead policies as presented here show themselves to be competent ecologically enlightened managers, able to anticipate and plan for the ecological responses of the pest to management actions, but if they are to anticipate the effects of evolution upon the focal population a more refined policy may be required (Ashley et al. 2003, Brown and Staňková 2017).

Look‐ahead policies combined releases with insecticidal sprays to achieve suppression in spite of the damage incurred by the heterozygous offspring resulting from matings between wild‐type females and transgenic males. Indeed, policies with longer planning horizons $H=\left\{2,3\right\}$ tended to make greater use of transgenic releases than insecticide. That is, by making fewer, better‐timed applications of insecticide, look‐ahead policies with longer planning horizons were able to attain a better outcome than equivalent policies with shorter horizons. Such policies are better able to resolve the downstream effects of a given action, which is especially useful with regard to male‐selecting transgenic releases, the benefits of which accrue across two temporally staggered phases. First, there is the initial suppression benefit received in the form of the removal of females heterozygous for the male‐selecting allele from the juvenile population. Second, males heterozygous for the male‐selecting allele can survive to maturity and potentially confer the allele upon their own offspring. This further reduces the future reproductive potential of the population, introgressing alleles for insecticide susceptibility and increasing the efficacy of a subsequent insecticide application (Alphey et al. 2007, 2009, Harvey‐Samuel et al. 2015, Leftwich et al. 2016, Alphey and Bonsall 2017, Zhou et al. 2018). By, at least partially, recognizing these benefits, look‐ahead policies were able to incorporate releases into their action sequences, reducing their dependency upon insecticide, an ecologically desirable outcome given the controversy surrounding pesticides and the environmental consequences of their overuse (Guedes et al. 2016).

Look‐ahead policies were adept at incorporating differences between early‐ and late‐acting male‐selecting releases and changes in juvenile survivorship into their decision making as these were factors with demographic consequences that could be observed within the bounds of the planning horizon. In contrast, they were mediocre resistance managers in spite of even the lowest initial resistance allele frequency used in the simulations $\left(r_{0}=0.3\right)$ being great enough to be considered problematic if observed under field conditions (Tabashnik and Carrière 2017). As such, these look‐ahead policies were reactionary managers, acting to reduce resistance only when it reduced the efficacy of sprays to the extent that it prevented adequate population suppression. Two major features of the current model framework impede the scope for look‐ahead policies to manage resistance. First, the emphasis upon cost minimization entails that “best practice” with respect to the objective function is likely to feature a non‐trivial level of resistance (Brown and Staňková 2017). Second, even the longest planning horizons used here are likely too short to offer sufficient information to the model regarding the future costs of resistance (Ashley et al. 2003, Brown and Staňková 2017). If look‐ahead policies are to be useful as a predictive tool in combining pest management scenarios with an evolutionary component, then their aptitude for accommodating detail‐rich problems will need to be linked to a capacity to anticipate consequences that lie beyond the planning horizon. These approaches possess the valuable capacity to include information relevant to the evolution of resistance such as the presence of fitness costs (to both transgenes and resistance alleles; Gassmann et al. 2009, Zhou et al. 2018), how fitness costs manifest (which may have demographic consequences as well as evolutionary ones; Guedes et al. 2016, Hackett and Bonsall 2016), or the extent to which the pest may be pre‐adapted to toxins (ffrench‐Constant and Bass 2017). However, their limited ability to reconcile the short‐term ecological effects and long‐term evolutionary effects of actions restricts the extent to which this information can be used effectively and biases them toward management practices that anticipate demographic responses but simply react to evolutionary responses (Ashley et al. 2003, Brown and Staňková 2017).

A fruitful area for future work may be the integration of look‐ahead policies with complementary methods that compensate for their constrained temporal perspective. A virtue of ADP is that each of the four major policy classes it encompasses circumvents the curses of dimensionality by distinct means (Powell 2011, 2014). Thus, the distinct policies are not mutually exclusive and they can be combined in a synergistic fashion to create so‐called hybrid polices when appropriate (Powell 2014, Powell and Meisel 2016). For example, cost function approximations (CFAs) are the simplest class of ADP policy. They operate by appending a correction term, which has been explicitly designed to either incentivize or discourage particular behaviors or outcomes, to the function we seek to optimize (Powell 2014, Powell and Meisel 2016). A simple hybrid policy could be constructed by introducing a CFA into Eq. (16) as a third term where it would act as a penalty term and devalue actions that allow the frequency of resistance to exceed some specified threshold.

A properly constructed hybrid policy could also serve as a means of introducing greater realism into the model structure. We utilized a simple framework for pest demography and for both the timing and implementation of actions in this model. However, in doing so we omitted numerous details relevant to the integration of sterile insect releases (transgenic or otherwise) and insecticides in reality. While such details would complicate the model, they may also provide an opportunity to showcase the versatility and applicability of these methods. In particular, rather having the freedom to select a release ratio, most sterile insect release programs would fix the magnitude and scheduling of releases for the season. Assuming that the magnitude of the release is appropriate then, as the pest population declines, the efficacy of each successive release is increased by the increasingly skewed ratio of sterile to wild‐type males (Barclay 2005). While the immediate suppression potential of male‐selecting transgenic releases is less than that of conventional sterile insects, they would also benefit from a similar increase in efficacy as the target population declines.

Using a hybrid policy, it may be possible to account for these structural constraints while simultaneously relieving the look‐ahead policy of the burden of reconciling two conflicting timescales. Specifically, a policy function approximation (PFA) could be useful in this context. PFA's are distinguished from other ADP policies in that they do not make a direct attempt to address the focal optimization problem. Instead, a parametric function describing a fixed decision rule is specified and the optimization problem shifts to identifying the parameter values that enable this rule to return the best valued action sequences (Powell 2014, Powell and Meisel 2016). For the current context, a PFA could be developed that fixes the scheduling of insecticide applications for a season such that some end of season benefit (e.g., yield) is maximized. The PFA could additionally define constraints that disallow spraying during certain intervals, such as the closing weeks of a season prior to harvest. It would then be possible to specify a look‐ahead policy that operates within the constraints of the spraying regime specified by the PFA to jointly determine the magnitude and frequency of releases for each season (e.g., release 20,000 insects every second week) such that the risk of insecticide resistance arising within a chosen timeframe is minimized. By fixing the spray schedule for each season and then shifting the look‐ahead policies focus to only one action per season, as opposed to one action per week as in the current model, then the look‐ahead policy would be able to plan over multiple seasons (e.g., with a planning horizon of *H* = 3 seasons as opposed to *H* = 3 weeks). Thus, such a problem formulation could provide the look‐ahead policy with the perspective required to actively manage the evolution of resistance.

Another route to improving the realism of the model would be to attempt to account for some of the uncertainties inherent in the management of agricultural insect pests. Many variables that we treated as known, such as the abundance of each pest life stage and the prevalence of resistance are, in reality, unobservable and would need to be inferred from, for example, monitoring data. Where key variables, parameters or processes for a system we seek to optimize are unobservable, or where important parameters are non‐stationary, adaptive management methods can be employed (Chadès et al. 2017). Adaptive management is a means of approaching the optimization of systems that are subject to structural uncertainty, which proceeds by recursively utilizing the difference between the anticipated and observed outcomes of actions to learn about key properties of the system that are not directly observable (Boettiger et al. 2015, Chadès et al. 2017). In this way, adaptive management takes an active approach to the control of complex systems by simultaneously identifying actions that would be expected to perform well, subject to current best estimates of hidden parameters and underlying processes, while also updating these estimates to improve future decisions (Chadès et al. 2017). As look‐ahead policies are structurally agnostic, they can accommodate updates to parameter values or even to the functional forms of the equations that underpin the model, which adaptive management requires.

Thus, look‐ahead policies, and ADP methods more generally, present the enticing opportunity of being able to consider management problems of a complexity that is more representative of those that would be encountered in reality at the expense of guaranteed optimality. At present, these methods may be best suited for the theoretical exploration of how the idiosyncratic complexities of a particular pest or environment could stand to influence best practice. Certainly, the slow runtime of look‐ahead policies for highly detailed problems likely precludes their use for on‐the‐spot decision making. But, in a less immediate context, where hours or days of computation time are not begrudged, they could be extremely valuable as a strategic tool for considering the commonalities and differences in suggested action sequences between diverse realistic management scenarios, potentially informed by available forecasts.

## Data Availability

The R code generated by this project has been uploaded to the Open Science Framework: https://osf.io/ja52e/

## References

[eap1851-bib-0001] Alphey, N. , and M. B. Bonsall . 2017 Genetics‐based methods for agricultural insect pest management. Agricultural and Forest Entomology 20:131–140. 29937693 10.1111/afe.12241 PMC5993313

[eap1851-bib-0002] Alphey, N. , P. G. Coleman , C. A. Donnelly , and L. Alphey . 2007 Managing insecticide resistance by mass release of engineered insects. Journal of Economic Entomology 100:1642–1649. 17972643 10.1603/0022-0493(2007)100[1642:mirbmr]2.0.co;2

[eap1851-bib-0003] Alphey, N. , M. B. Bonsall , and L. Alphey . 2009 Combining pest control and resistance management: synergy of engineered insects with Bt crops. Journal of Economic Entomology 102:717–32. 19449654 10.1603/029.102.0233

[eap1851-bib-0004] Ashley, M. V. , M. F. Willson , O. R. W. Pergams , D. J. O'Dowd , S. M. Gende , and J. S. Brown . 2003 Evolutionarily enlightened management. Biological Conservation 111:115–123.

[eap1851-bib-0005] Barclay, H. 2005 Mathematical models for the use of sterile insects Pages 147–174 *in* Dyck V., Hendrichs J., and Robinson A. S., editors. Sterile insect technique principles and practice in area‐wide integrated pest management. First edition Springer, Dordrecht, The Netherlands.

[eap1851-bib-0006] Boettiger, C. , M. Mangel , and S. Munch . 2015 Avoiding tipping points in fisheries management through Gaussian process dynamic programming. Proceedings of the Royal Society B 282:8–11. 10.1098/rspb.2014.1631 PMC4308990 25567644

[eap1851-bib-0007] Bourtzis, K. , R. S. Lees , J. Hendrichs , and M. J. B. Vreysen . 2016 More than one rabbit out of the hat: radiation, transgenic and symbiont‐based approaches for sustainable management of mosquito and tsetse fly populations. Acta Tropica 157:115–130. 26774684 10.1016/j.actatropica.2016.01.009

[eap1851-bib-0008] Brown, J. S. , and K. Staňková . 2017 Game theory as a conceptual framework for managing insect pests. Current Opinion in Insect Science 21:26–32. 28822485 10.1016/j.cois.2017.05.007

[eap1851-bib-0009] Chadès, I. , S. Nicol , T. M. Rout , M. Péron , Y. Dujardin , J. B. Pichancourt , A. Hastings , and C. E. Hauser . 2017 Optimization methods to solve adaptive management problems. Theoretical Ecology 10:1–20.

[eap1851-bib-0010] Clark, C. W. , and M. Mangel . 2000 Dynamic state variable models in ecology: methods and applications. Oxford University Press, Oxford, UK.

[eap1851-bib-0011] ffrench‐Constant, R. H. , and C. Bass . 2017 Does resistance really carry a fitness cost? Current Opinion in Insect Science 21:39–46. 28822487 10.1016/j.cois.2017.04.011 PMC5972224

[eap1851-bib-0012] Garcia, A. G. , C. P. Ferreira , F. L. Cônsoli , and W. A. C. Godoy . 2016 Predicting evolution of insect resistance to transgenic crops in within‐field refuge configurations, based on larval movement. Ecological Complexity 28:94–103.

[eap1851-bib-0013] Gassmann, A. J. , Y. Carrière , and B. E. Tabashnik . 2009 Fitness costs of insect resistance to Bacillus thuringiensis. Annual Review of Entomology 54:147–63. 10.1146/annurev.ento.54.110807.090518 19067630

[eap1851-bib-0014] Guedes, R. N. C. , G. Smagghe , J. D. Stark , and N. Desneux . 2016 Pesticide‐induced stress in arthropod pests for optimized integrated pest management programs. Annual Review of Entomology 61:43–62. 10.1146/annurev-ento-010715-023646 26473315

[eap1851-bib-0015] Hackett, S. C. , and M. B. Bonsall . 2016 Type of fitness cost influences the rate of evolution of resistance to transgenic Bt crops. Journal of Applied Ecology 53:1391–1401. 27708457 10.1111/1365-2664.12680 PMC5026168

[eap1851-bib-0016] Hackett, S. C. , and M. B. Bonsall . 2018 Management of a stage‐structured insect pest: an application of approximate optimization. Ecological Applications 28:938–952. 29431888 10.1002/eap.1700

[eap1851-bib-0017] Harvey‐Samuel, T. , et al. 2015 Pest control and resistance management through release of insects carrying a male‐selecting transgene. BMC Biology 13:49. 26179401 10.1186/s12915-015-0161-1 PMC4504119

[eap1851-bib-0018] Harvey‐Samuel, T. , T. Ant , and L. Alphey . 2017 Towards the genetic control of invasive species. Biological Invasions 19:1683–1703. 28620268 10.1007/s10530-017-1384-6 PMC5446844

[eap1851-bib-0019] Leftwich, P. T. , M. Bolton , and T. Chapman . 2016 Evolutionary biology and genetic techniques for insect control. Evolutionary Applications 9:212–230. 27087849 10.1111/eva.12280 PMC4780389

[eap1851-bib-0020] Marescot, L. , G. Chapron , I. Chades , P. L. Fackler , C. Duchamp , E. Marboutin , and O. Gimenez . 2013 Complex decisions made simple: a primer on stochastic dynamic programming. Methods in Ecology and Evolution 4:872–884.

[eap1851-bib-0021] Miller, N. J. , and T. W. Sappington . 2017 Role of dispersal in resistance evolution and spread. Current Opinion in Insect Science 21:68–74. 28822491 10.1016/j.cois.2017.04.005

[eap1851-bib-0022] Mitchell, P. D. , and D. Onstad . 2014 Valuing pest susceptibility to control Pages 25–53 *in* Onstad D., editor. Insect resistance management: biology, economics and prediction, Second edition Academic Press, Cambridge, Massachusetts, USA.

[eap1851-bib-0023] Nicol, S. , and I. Chadès . 2011 Beyond stochastic dynamic programming: a heuristic sampling method for optimizing conservation decisions in very large state spaces. Methods in Ecology and Evolution 2:221–228.

[eap1851-bib-0024] Nicol, S. C. , I. Chadès , S. Linke , and H. P. Possingham . 2010 Conservation decision‐making in large state spaces. Ecological Modelling 221:2531–2536.

[eap1851-bib-0025] Powell, W. B. 2011 Approximate dynamic programming. Second edition John Wiley & Sons, Hoboken, New Jersey, USA.

[eap1851-bib-0026] Powell, W. B. 2014 Clearing the jungle of stochastic optimization Pages 109–137 *in* Newman A. and Leung J., editors. Bridging data and decisions. INFORMS, Cantonville, Maryland, USA.

[eap1851-bib-0027] Powell, W. B. , and S. Meisel . 2016 Tutorial on stochastic optimization in energy—Part II: an energy storage illustration. IEEE Transactions on Power Systems 31:1468–1475.

[eap1851-bib-0028] REX Consortium . 2013 Heterogeneity of selection and the evolution of resistance. Trends in Ecology & Evolution 28:110–118. 23040463 10.1016/j.tree.2012.09.001

[eap1851-bib-0029] Sudo, M. , D. Takahashi , D. A. Andow , Y. Suzuki , and T. Yamanaka . 2018 Optimal management strategy of insecticide resistance under various insect life histories: Heterogeneous timing of selection and interpatch dispersal. Evolutionary Applications 11:271–283. 29387161 10.1111/eva.12550 PMC5775500

[eap1851-bib-0030] Tabashnik, B. E. , and Y. Carrière . 2017 Surge in insect resistance to transgenic crops and prospects for sustainability. Nature Biotechnology 35:926–935. 10.1038/nbt.3974 29020006

[eap1851-bib-0031] Téllez‐Rodríguez, P. , B. Raymond , I. Morán‐Bertot , L. Rodríguez‐Cabrera , D. J. Wright , C. G. Borroto , and C. Ayra‐Pardo . 2014 Strong oviposition preference for Bt over non‐Bt maize in *Spodoptera frugiperda* and its implications for the evolution of resistance. BMC Biology 12:48. 24935031 10.1186/1741-7007-12-48 PMC4094916

[eap1851-bib-0032] Watkinson‐Powell, B. , and N. Alphey . 2017 Resistance to genetic insect control: modelling the effects of space. Journal of Theoretical Biology 413:72–85. 27816677 10.1016/j.jtbi.2016.10.014 PMC5177727

[eap1851-bib-0033] Yakob, L. , and M. B. Bonsall . 2009 Importance of space and competition in optimizing genetic control strategies. Journal of Economic Entomology 102:50–57. 19253617 10.1603/029.102.0108

[eap1851-bib-0034] Zhou, L. , N. Alphey , A. S. Walker , L. M. Travers , F. Hasan , N. I. Morrison , M. B. Bonsall , and B. Raymond . 2018 Combining the high‐dose/refuge strategy and self‐limiting transgenic insects in resistance management—A test in experimental mesocosms. Evolutionary Applications 11:727–738. 29875814 10.1111/eva.12573 PMC5979637

