# Supplementary material for: Insect pest control, approximate dynamic programming, and the management of the evolution of resistance
Source: Ecol Appl. 2019 Feb 12;29(2):e01851. doi: 10.1002/eap.1851 (PMC6850168; doi:10.1002/eap.1851)
Supplement: Supplementary file 1 [file EAP-29-na-s001.pdf]

Supporting Information. Hackett, S. C. and M. B. Bonsall. 2019. Insect pest control, approximate dynamic programming and the management of the evolution of resistance. *Ecological Applications.*

Appendix S1

When juveniles are robust ($\mu_j = 0.05$) there is a reduction in the frequency of transgenic releases and a greater emphasis on insecticide application. Consequently, resistance allele frequencies tend to be greater than those observed for the default population as illustrated by Figure S1 which presents the resistance allele trajectories observed for late acting releases. The increased preference for spraying applies independently of the type of transgenic release and affects all considered policies, even those with longer planning horizons (*e.g.* $H = 3$, Figure S2). However, the general trends detailed for the default population ($\mu_j = 0.2$) still hold true for the robust population. For example, while sprays are applied more frequently by all policies, those with longer planning horizons still deploy sprays less frequently than policies with shorter horizons for given values of h and r_0. This shift in action preference is attributable to the sensitivity of the model to juvenile abundance and persistence. Juveniles inflict damage upon the crop but cannot be directly removed. This biases policies with short planning horizons against transgenic releases as they cannot adequately perceive the potential future benefits that may follow from temporarily accepting the presence of heterozygous larvae within the crop. Instead, they focus on spraying which yields immediate population suppression. In contrast, policies with longer horizons are better able to integrate releases and sprays and tend to favour few well-timed sprays interspersed with transgenic releases (*e.g.*Fig.1 & Fig.2). However, increasing juvenile survival indirectly increases the amount of damage any given juvenile population can inflict. Thus, the threat posed by even a small juvenile population is increased which promotes spraying, the action which produces the greatest immediate reduction in juvenile recruitment, whilst narrowing the range of circumstances under which releases are selected.

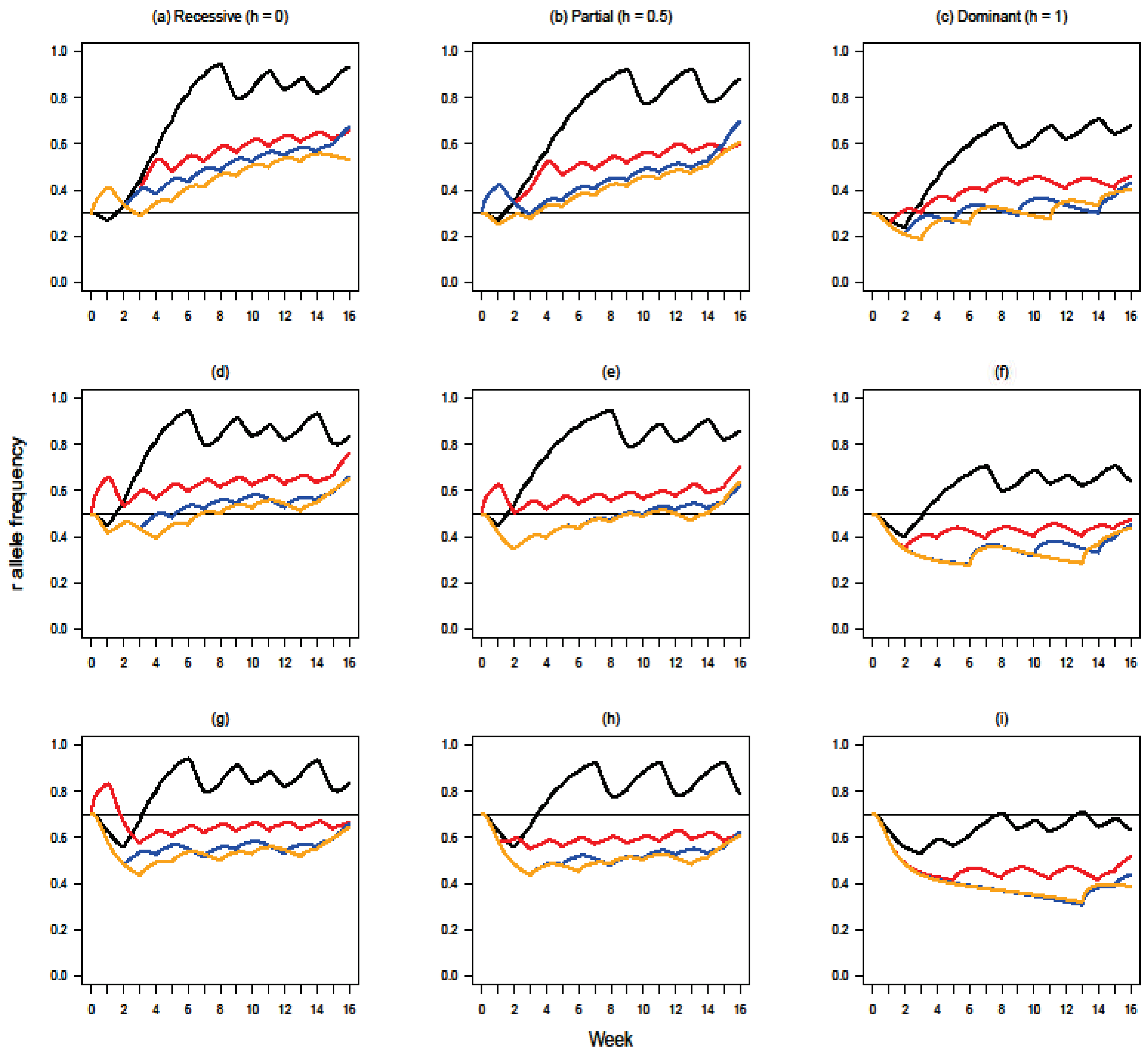

Figure S1: As for Figure 1 but with a reduced instantaneous juvenile mortality rate, $\mu_j = 0.05$.

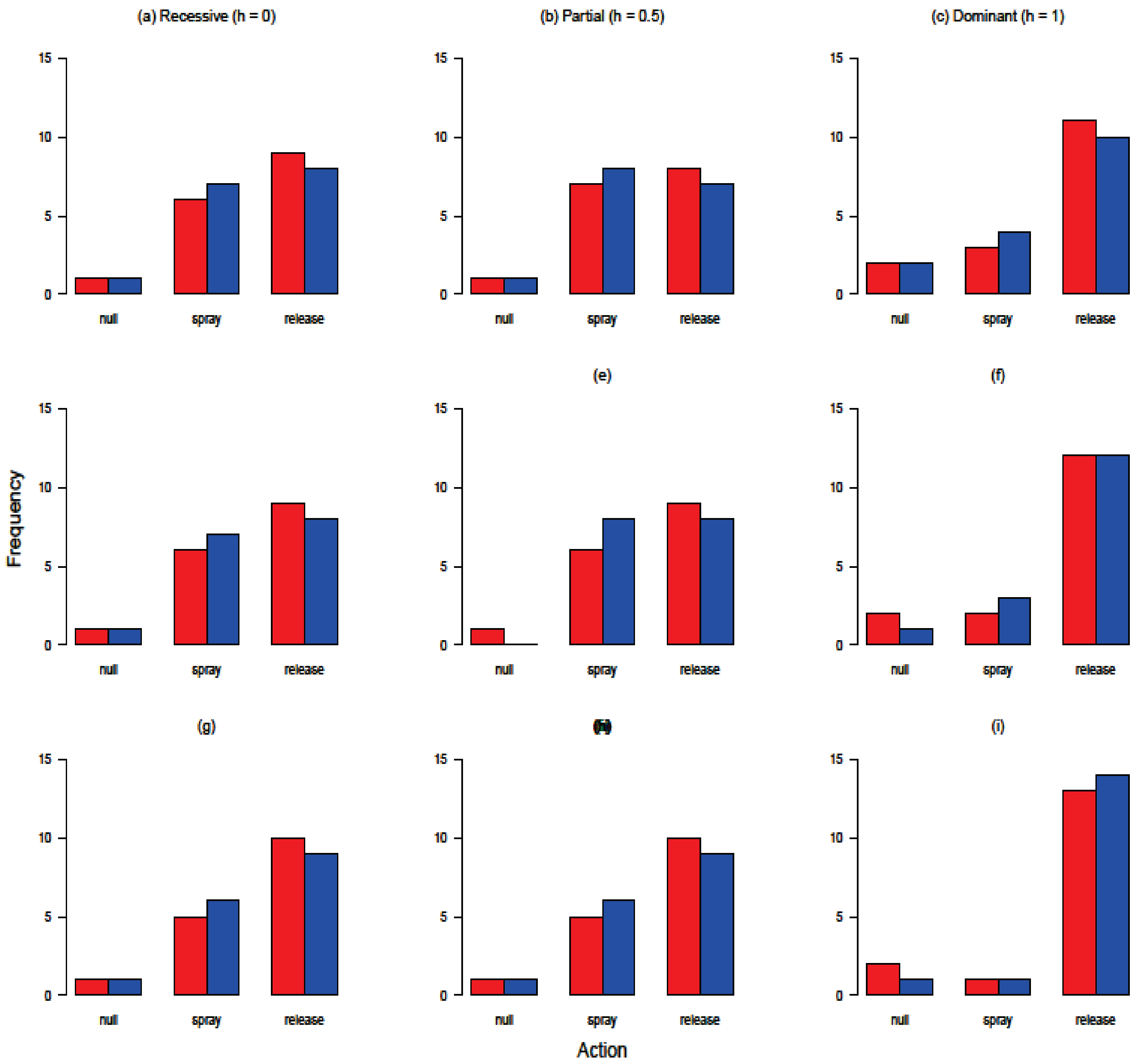

Figure S2: As for Figure 3 but with a reduced instantaneous juvenile mortality rate, $\mu_j = 0.05$.
